# An Alternative Technique to Strut Change in Hexapod Circular External Fixator

**DOI:** 10.5704/MOJ.2111.029

**Published:** 2021-11

**Authors:** M Rao, K Jamil, R Tasarib

**Affiliations:** 1 Department of Orthopaedics, Hospital Serdang, Kajang, Malaysia; 2 Department of Orthopaedics and Traumatology, Universiti Kebangsaan Malaysia, Kuala Lumpur, Malaysia

Dear editors,

Hexapod circular external fixators such as the Taylor Spatial Frame [TSF, Smith and Nephew, Memphis, USA] and the Ortho-SUV [OSF, Pitkar-Orthotools, Pune, India] are increasingly being used in the correction of limb deformities^1,2^. The device comprises of six struts of various lengths between the two rings to accommodate a multitude of deformities. It can correct three-dimensional deformities simultaneously using the concept of a virtual hinge, guided by a web-based programme.

The patient must manipulate the struts to a specified length during the treatment programme to achieve a gradual correction. The strut lengths may reach a limit, and it may be necessary to change the struts at certain intervals of the correction. To prevent instability during the change of a strut, the use of a seventh strut has been recommended^3^. Correct positioning of this seventh strut is crucial, as malpositioning may result in frame collapse during the strut change. A catastrophic collapse during the change of struts may lead to the severe displacement of bone segments across the osteotomy site^4^.

However, the placement of a temporary strut may be hindered by the ring configuration and the presence of Kirshner wires or half pins. In this situation, a threaded rod with a conical washer was proposed, but the technical details were not described^3^. Therefore, we propose an alternative technique of using a 'triple hinge pillar' (THP) during the change of strut.

**Fig 1:**
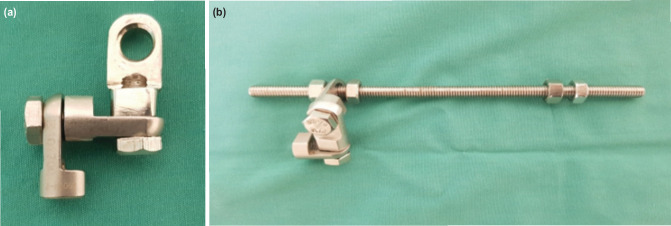
(a)The triple hinge unit which is made out of 3 hinges attached at right angle to each other. This configuration allows movement in 3-dimensional planes. (b) Triple-hinge unit which is attached to a threaded rod and hence named triple hinge pillar. The threaded rod of various lengths can be chosen depending on the length required.

A 'triple hinge' consists of three hinges attached at right angles of each other using a short bolt (Fig. 1a). This unit is then connected at a 90° angle to a threaded rod with two nuts to secure it in place (Fig. 1b).

**Fig 2:**
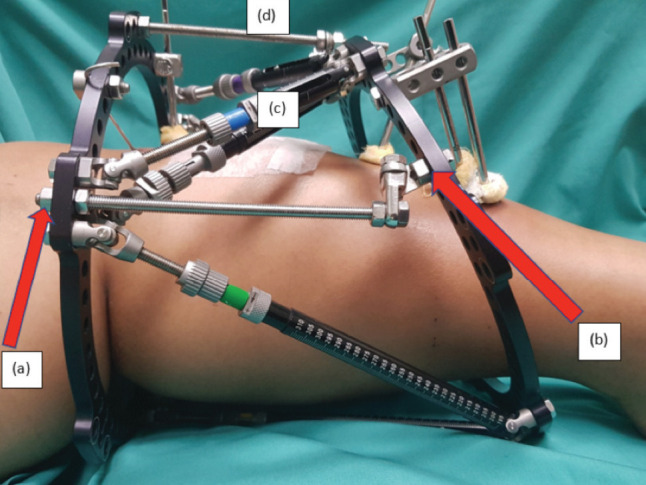
(a) Fixation of the triple hinge pillar begins with the application of the threaded rod onto 1 ring. (b) Next the loosened triple hinge unit is arranged to allow fixation onto the next ring. The strut (c) can now be removed. Another triple hinge unit (d) can also be added for multiple struts change.

Before removal of the strut, we recommend the application of THP at the region of the strut to be removed. Any available holes on the ring may be used. First, all connections of the triple-hinge unit are loosened to allow free movement. The threaded rod is then attached to one ring of the frame (Fig. 2). Next, the THP is positioned to allow attachment onto the adjacent ring. Once the pillar has been fixed in the optimal position, all connections are tightened using a standard size-10 wrench. The strut may now be removed and replaced as needed. At this point, the temporary THP maintains the stability of the frame. More than one may be attached to the frame if additional strut change is required. Once the change of strut is made and the replacement strut is positioned at the desired length, then the THP may be safely removed.

**Fig 3:**
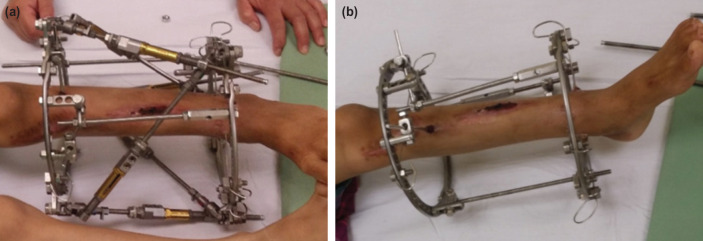
(a) Triple hinge pillar attached on the SUV frame prior to removal of struts. (b) All struts have been removed and replaced with three triple hinge pillars.

We have shown a simple technique to stabilise the frame during a strut change temporarily. THP can also be used to remove SUV struts for transformation into a modular frame following completion of deformity correction (Fig. 3a, 3b). The unit has three axes of rotation which allow fitting into any ring configuration with ease. The length of the threaded rod can be chosen depending on the configuration. Multiple struts change is faster but is safe with this technique.
